# ctDNA and residual cancer burden are prognostic in triple-negative breast cancer patients with residual disease

**DOI:** 10.1038/s41523-023-00512-7

**Published:** 2023-03-06

**Authors:** Shane R. Stecklein, Bruce F. Kimler, Rachel Yoder, Kelsey Schwensen, Joshua M. Staley, Qamar J. Khan, Anne P. O’Dea, Lauren E. Nye, Manana Elia, Jaimie Heldstab, Trisha Home, Stephen Hyter, Kamilla Isakova, Harsh B. Pathak, Andrew K. Godwin, Priyanka Sharma

**Affiliations:** 1 Department of Radiation Oncology, University of Kansas Medical Center, Kansas City, KS USA; 2 Department of Pathology and Laboratory Medicine, University of Kansas Medical Center, Kansas City, KS USA; 3 Department of Cancer Biology, University of Kansas Medical Center, Kansas City, KS USA; 4 The University of Kansas Cancer Center, University of Kansas Medical Center, Kansas City, KS USA; 5 Division of Medical Oncology, Department of Internal Medicine, University of Kansas Medical Center, Kansas City, KS USA

**Keywords:** Breast cancer, Prognostic markers

## Abstract

Triple-negative breast cancer (TNBC) patients with residual disease (RD) after neoadjuvant systemic therapy (NAST) are at high risk for recurrence. Biomarkers to risk-stratify patients with RD could help individualize adjuvant therapy and inform future adjuvant therapy trials. We aim to investigate the impact of circulating tumor DNA (ctDNA) status and residual cancer burden (RCB) class on outcomes in TNBC patients with RD. We analyze end-of-treatment ctDNA status in 80 TNBC patients with residual disease who are enrolled in a prospective multisite registry. Among 80 patients, 33% are ctDNA positive (ctDNA+) and RCB class distribution is RCB-I = 26%, RCB-II = 49%, RCB-III = 18% and 7% unknown. ctDNA status is associated with RCB status, with 14%, 31%, and 57% of patients within RCB-I, -II, and -III classes demonstrating ctDNA+ status (*P* = 0.028). ctDNA+ status is associated with inferior 3-year EFS (48% vs. 82%, *P* < 0.001) and OS (50% vs. 86%, *P* = 0.002). ctDNA+ status predicts inferior 3-year EFS among RCB-II patients (65% vs. 87%, *P* = 0.044) and shows a trend for inferior EFS among RCB-III patients (13% vs. 40%, *P* = 0.081). On multivariate analysis accounting for T stage and nodal status, RCB class and ctDNA status independently predict EFS (HR = 5.16, *P* = 0.016 for RCB class; HR = 3.71, *P* = 0.020 for ctDNA status). End-of-treatment ctDNA is detectable in one-third of TNBC patients with residual disease after NAST. ctDNA status and RCB are independently prognostic in this setting.

## Introduction

Triple-negative breast cancer (TNBC) accounts for 15% of all breast cancers and is associated with higher rates of recurrence and death compared to non-triple-negative breast cancers^1^. Neoadjuvant systemic therapy (NAST) is commonly employed in TNBC, and residual disease after NAST is associated with a high risk of recurrence and death^2,3^. The addition of adjuvant chemotherapy with capecitabine in TNBC patients with residual disease has been shown to improve outcomes, though many patients still experience disease recurrence despite the addition of adjuvant chemotherapy, and this approach may lead to overtreatment in some patients since not all patients with residual disease experience a recurrence^4,5^. Thus, tools to further stratify the risk of recurrence in patients with residual disease can optimize the utilization of available adjuvant therapy and improve the efficiency of clinical trials investigating novel agents in this setting. One of these risk-stratifying tools, the residual cancer burden (RCB) classification, quantitates the extent of residual disease in the breast and axillary lymph nodes following neoadjuvant chemotherapy and adds prognostic value to the binary assessment of pathological complete response vs. residual disease in predicting long-term survival^6,7^.

Recent studies have shown that detectable cell-free circulating tumor DNA (ctDNA) after NAST is prognostic in TNBC patients with residual disease^8–10^. Because both RCB and ctDNA status are prognostic among patients with TNBC with residual disease, assessing the combined impact of both is of interest. Most TNBC patients with residual disease will receive adjuvant radiotherapy and/or chemotherapy, and recent work has shown that adjuvant therapy may influence ctDNA status in patients with residual disease^11^. Thus, ctDNA status at the completion of all definitive treatment is likely to be a good indicator of the efficacy of currently available curative therapy. The impact of end-of-treatment (EOT) ctDNA status on long-term prognosis and its ability to complement the prognostic utility of the RCB classification has not been investigated. The purpose of this study was to investigate the combined impact of EOT ctDNA status and RCB class on outcomes in TNBC patients with residual disease. We hypothesized that RCB class and ctDNA status may provide complementary prognostic information.

We detected ctDNA in 33% of TNBC patients with residual disease after NAST. ctDNA status is associated with RCB class, though these two biomarkers provide complementary but not completely overlapping prognostic information, particularly in patients with RCB-II disease. These findings, if confirmed in additional studies, could provide insights into the role of ctDNA in identifying patients who are most likely to benefit from adjuvant treatment intensification. Because of the complementary prognostic information provided by RCB class and ctDNA status, we suggest that future residual disease adjuvant therapy trials for TNBC patients consider both biomarkers for patient selection/stratification.

## Results

### Patient characteristics and univariate analysis of clinicopathologic features and survival

81 patients with residual disease and an available EOT plasma sample for ctDNA analysis were identified (see REMARK, Supplementary Fig. 1A). ctDNA sequencing was unsuccessful in one patient, so the final study cohort includes 80 patients. There was no difference in baseline disease characteristics between the EOT ctDNA available cohort (*N* = 80) and all patients with residual disease (*N* = 268) (data not shown). Similarly, there was no difference in survival outcomes between ctDNA-available patients and all patients with residual disease (3-year EFS 70% for ctDNA-available cohort vs. 70% for all residual disease patients, *P* = 0.782; 3-year OS 74% for ctDNA-available cohort vs. 77% for all residual disease patients, *P* = 0.520).

**Fig. 1:**
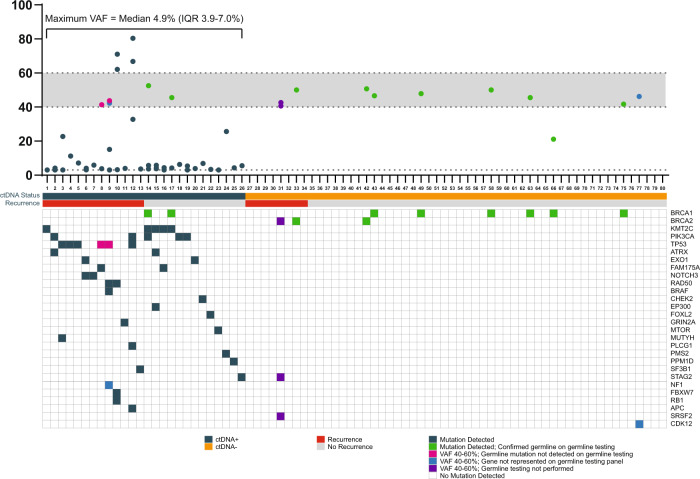
Mutations detected in ctDNA are shown for individual patients, with variant allele frequencies and germline testing results. Patient-level ctDNA mutations and variant allele thresholds.

We evaluated lower boundary VAF thresholds from 1% to 5% (Supplementary Fig. 1B) and found that a lower boundary of 3% gave the best discrimination for EFS (Supplementary Fig. 1C), so this threshold was used to define ctDNA status. Thirteen patients (16%) had one or more mutations detected at 40–60% VAF (Fig. 1). Ten of these patients had a cfDNA mutation in *BRCA1* or *BRCA2*, and 9/10 were confirmed to be germline from clinical genetic testing reports. The remaining patient with a detectable *BRCA2* ctDNA mutation did not undergo germline genetic testing and had co-existing 40–60% VAF cfDNA mutations in *SRSF2* and *STAG2*. One patient had a *BRCA1* mutation with a VAF of 21%, but this was confirmed germline on clinical genetic testing, so this patient was classified as ctDNA−. Two patients had 40–60% VAF cfDNA mutations in genes that were not represented on their clinical genetic testing panels (*NF1* and *CDK12*); one of these had co-existing cfDNA mutations that fell within the ≥3% and <40% range and therefore was classified as ctDNA+. Two patients had 40–60% VAF cfDNA mutations in *TP53*, and these mutations were not detected on clinical genetic testing panels that included *TP53*. Both of these patients had co-existing low-frequency cfDNA mutations that rendered them ctDNA+, so this did not impact their status. Using the ≥3% but <40% or >60% threshold, the median per-patient maximum VAF (excluding confirmed germline mutations) was 4.9% with an interquartile range of 3.9–7.0% (Fig. 1). Baseline patient and tumor characteristics for the study cohort are shown in Table 1: 14% of patients were Black, 40% of patients had node-positive disease, and 24% of patients had stage III disease at presentation. Median time from completion of curative treatment to EOT plasma sample collection was 113 days (range 0–180 days; Supplementary Fig. 1D), and ctDNA was detectable in 26/80 (33%) patients (Fig. 1). The frequency of ctDNA+ status did not differ by time to sample collection when analyzing in six 30-day bins (*P* = 0.300) or when partitioning by median (*P* = 0.635) (Supplementary Fig. 1D). Patients with detectable ctDNA (ctDNA+) had a higher TNM stage (*P* = 0.029), and this was driven by a trend toward higher rates of lymph node involvement (*P* = 0.079). The rate of ctDNA positivity was associated with RCB class, with ctDNA positivity rates of 14%, 31%, and 57% in RCB-I, -II, and -III classes, respectively (*P* = 0.028; Table 2). Distribution of age, menopausal status, race, ethnicity, germline *BRCA1/2* mutation status, T stage, NAST regimen, receipt of neoadjuvant immunotherapy, and receipt of adjuvant systemic therapy were not significantly different between ctDNA+ and ctDNA− patients. 49% of patients received adjuvant chemotherapy, and no patient received adjuvant immunotherapy (Table 1).

**Table 1:** Baseline patient and tumor characteristics.

	All Patients (*N* = 80)		ctDNA-Negative (*N* = 54)		ctDNA-Positive (*N* = 26)		*P*
Median age (range)	48	25–85	48	25–70	52	28–85	0.537
*Menopausal status*							0.290
Pre	38	48%	28	52%	10	38%	
Post	40	50%	25	46%	15	58%	
Unknown^a^	2	3%	1	2%	1	4%	
*Race*							0.082
White	64	80%	45	83%	19	73%	
Black	11	14%	8	15%	3	12%	
Other	5	6%	1	2%	4	15%	
*Ethnicity*							0.098
Not Hispanic	76	95%	53	98%	23	88%	
Hispanic	4	5%	1	2%	3	12%	
*Germline BRCA1/2 mutation*							0.743
No	62	77%	42	78%	20	77%	
Yes	11	14%	8	15%	3	12%	
Unknown^a^	7	9%	4	7%	3	12%	
*T stage*							0.128
1	13	16%	10	19%	3	12%	
2	44	55%	33	59%	12	46%	
3	19	24%	8	17%	10	38%	
4	4	5%	3	6%	1	4%	
*Nodal status*							0.079
Negative	48	60%	36	67%	12	46%	
Positive	32	40%	18	33%	14	54%	
*TNM stage*							0.029
I	11	14%	8	15%	3	12%	
II	50	63%	38	70%	12	46%	
III	19	24%	8	15%	11	42%	
*Neoadjuvant chemotherapy regimen*							0.285
Anthracycline ± taxane	16	20%	8	15%	8	31%	
Anthracycline/taxane/platinum	13	16%	9	17%	4	15%	
Taxane/platinum	49	61%	36	67%	13	50%	
Taxane	2	3%	1	2%	1	4%	
*Neoadjuvant immunotherapy*							0.644
No	61	76%	42	78%	19	73%	
Yes	19	24%	12	22%	7	27%	
*Surgery type*							0.048
Lumpectomy	24	30%	29	37%	4	15%	
Mastectomy	56	70%	34	63%	22	85%	
*Adjuvant radiotherapy*							0.924
Yes	59	74%	40	74%	19	73%	
No	21	26%	14	26%	7	27%	
*Adjuvant chemotherapy*							0.877
Yes	39	49%	26	48%	13	50%	
No	41	51%	28	52%	13	50%	

Baseline patient and tumor characteristics.

*P* for continuous variables is Mann–Whitney *U* test. *P* for categorical comparisons is chi-square or Fisher–Freeman–Halton Exact test.

^a^Patients with unknown status were excluded from statistical comparison.

**Table 2:** ctDNA status by RCB class.

	All Patients (*N* = 80)		ctDNA-Negative (*N* = 54)		ctDNA-Positive (*N* = 26)		*P*
*RCB class*							0.028
I	21	26%	18	33%	3	12%	
II	39	49%	27	50%	12	46%	
III	14	18%	6	11%	8	31%	
Unknown^a^	6	7%	3	6%	3	12%	

Association between ctDNA status and RCB class.

*P* is Fisher–Freeman–Halton Exact test.

^a^Patients with unknown status were excluded from the statistical comparison.

### ctDNA status and RCB class are associated with survival

**Fig. 2:**
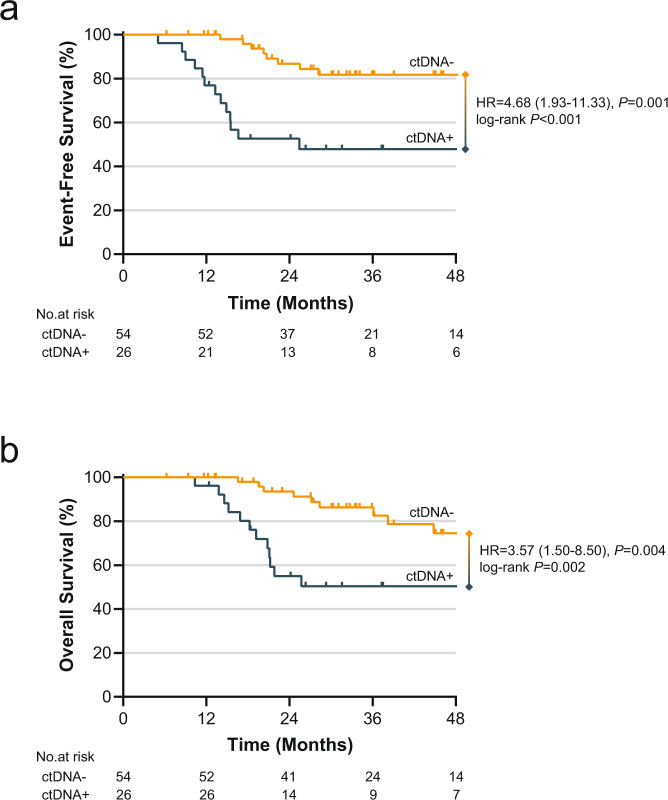
Survival by ctDNA status. **a** EFS among ctDNA− and ctDNA+ patients. **b** OS among ctDNA− and ctDNA+ patients. *P* value associated with HR is Cox univariate regression. Log-rank *P* is also provided.

At a median follow-up of 31 months, there have been 21 events (4 local only, 17 distant) and 21 deaths, and the estimated 3-year EFS and OS for all patients were 70% and 74%, respectively. ctDNA+ status was significantly associated with inferior EFS (3-year EFS 48% in ctDNA+ vs. 82% in ctDNA−, log-rank *P* < 0.001; HR = 4.68, 95% CI 1.93–11.33, Cox proportional hazard *P* = 0.001) and OS (3-year OS 50% in ctDNA+ vs. 86% in ctDNA−, log-rank *P* = 0.002; HR = 3.57, 95% CI 1.50–8.50, Cox proportional hazard *P* = 0.004) (Fig. 2). There was no apparent association between the interval from surgery to ctDNA assessment and the interval from ctDNA assessment to EFS event in patients who experienced recurrence (Supplementary Fig. 1E). Among ctDNA+ patients who experienced an EFS event, the median time from ctDNA detection to the event was 4.7 months. Similarly, increasing RCB class was associated with significantly worse EFS and OS. Both 3-year EFS and OS for patients with RCB class I, II, and III were 100%, 79%, and 23%, respectively (log-rank *P* < 0.001 for both; Supplementary Fig. 2). On univariate analysis, higher presenting T stage (T3–T4 vs. T1–2), positive nodal status (node-positive vs. negative), and higher TNM stage (stage III vs. I) were also associated with significantly worse EFS and OS (Table 3).

**Table 3:** Univariate analysis for event-free and overall survival.

Variable	EFS			OS		
	HR	95% CI	*P*	HR	95% CI	*P*
Race						
White	1	–		1	–	
Black	0.62	0.14–2.69	0.523	0.64	0.15–2.76	0.546
Other	1.94	0.45–8.42	0.375	2.42	0.55–10.56	0.241
Ethnicity						
Non-Hispanic	1	–		1	–	
Hispanic	2.30	0.54–9.87	0.250	2.21	0.51–9.50	0.275
Germline *BRCA1/2* mutation						
Yes	1	–		1	–	
No	0.64	0.15–2.81	0.554	0.62	0.14–2.71	0.518
T stage						
1-2	1	–		1	–	
3-4	3.56	1.51–8.38	0.004	4.38	1.84–10.43	0.001
Nodal status						
Negative	1	–		1	–	
Positive	3.61	1.46–8.96	0.006	2.72	1.13–6.56	0.026
TNM stage						
I	1	–		1	–	
II	2.01	0.26–15.90	0.507	1.82	0.23–14.38	0.571
III	8.97	1.16–69.60	0.036	8.11	1.05–62.90	0.023
RCB class^a^						
II	1	–		1	–	
III	6.58	2.48–17.48	<0.001	3.95	1.51–10.31	0.005
Adjuvant chemotherapy						
Yes	1	–		1	–	
No	0.85	0.36–2.00	0.711	0.86	0.37–2.03	0.736
ctDNA status						
Negative	1	–		1	–	
Positive	4.68	1.93–11.33	0.001	3.57	1.50–8.50	0.004

Univariate analysis of EFS and OS based on clinicopathologic variables and ctDNA status.

*P* is from Cox proportional hazards regression.

^a^There were no EFS events in RCB-I patients.

### ctDNA complements RCB in prognosticating TNBC patients with residual disease

**Fig. 3:**
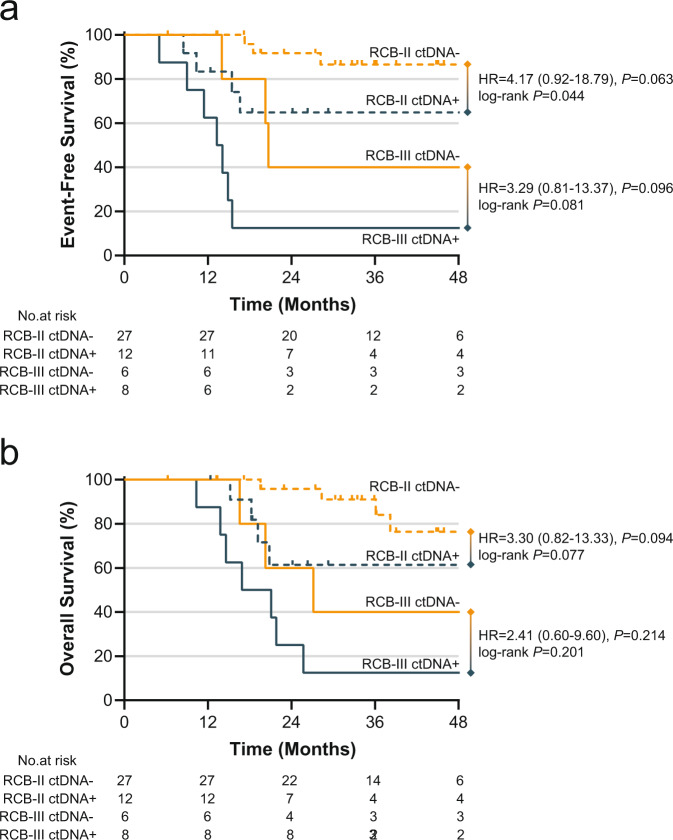
Survival by ctDNA status within RCB classes. **a** EFS among ctDNA− and ctDNA+ patients within RCB-II and RCB-III classes. **b** OS among ctDNA− and ctDNA+ patients within RCB-II and RCB-III classes. *P* value associated with HR is Cox univariate regression. Log-rank *P* is also provided.

Since RCB is a validated measure of recurrence risk after NAST, we sought to examine the utility of classifying patients by both RCB class and ctDNA status to determine if dual classification provided additional prognostic information. There were no EFS or OS events in patients with RCB-I, so the prognostic impact of ctDNA in the RCB-I group could not be evaluated (Supplementary Fig. 2). In patients with RCB-II disease, ctDNA+ status was significantly associated with inferior EFS (3-year EFS 65% in ctDNA+ vs. 87% in ctDNA−, log-rank *P* = 0.044; HR = 4.17, 95% CI 0.92–18.79, Cox proportional hazard *P* = 0.063) and a trend toward inferior OS (3-year OS 61% for ctDNA+ vs. 76% for ctDNA−, log-rank *P* = 0.077; HR = 3.30, 95% CI: 0.82–13.33, Cox proportional hazard *P* = 0.094). In patients with RCB-III disease, ctDNA+ status was also associated with a trend toward inferior EFS (3-year EFS 13% for ctDNA+ vs. 40% for ctDNA−, log-rank *P* = 0.081; HR = 3.29, 95% CI: 0.81–13.37, Cox proportional hazard *P* = 0.096) and a trend toward inferior OS (3-year OS 13% for ctDNA+ vs. 40% for ctDNA−, log-rank *P* = 0.201; HR = 2.41, 95% CI: 0.60–9.60, Cox proportional hazard *P* = 0.214) (Fig. 3). In a multivariate Cox regression analysis (including T stage, nodal status, RCB class, and ctDNA status), RCB class and ctDNA status remained prognostic for EFS (HR = 5.16, 95% CI: 1.36–19.52, *P* = 0.016 (RCB class); HR = 3.71, 95% CI: 1.23–11.21, *P* = 0.020 (ctDNA status)). For OS, RCB class remained prognostic (HR = 3.53, 95% CI: 1.10–11.35, *P* = 0.034) and ctDNA status showed a prognostic trend (HR = 2.59, 95% CI: 0.87–7.77, *P* = 0.089) (Table 4).

**Table 4:** Multivariate analysis for event-free and overall survival.

Variable	EFS			OS		
	HR	95% CI	*P*	HR	95% CI	*P*
T stage						
T1–T2	1	–		1	–	
T3–T4	1.03	0.30–3.58	0.964	1.61	0.52–5.00	0.409
Nodal status						
Negative	1	–		1	–	
Positive	1.09	0.28–4.23	0.897	0.89	0.26–3.10	0.858
RCB class^a^						
RCB-II	1	–		1	–	
RCB-III	5.16	1.36–19.52	0.016	3.53	1.10–11.35	0.034
ctDNA status						
Negative	1	–		1	–	
Positive	3.71	1.23–11.21	0.020	2.59	0.87–7.77	0.089

Multivariate analysis of EFS and OS based on clinicopathologic variables and ctDNA status.

*P* is from Cox proportional hazards regression.

^a^Multivariate analysis restricted to RCB-II/III patients, as there were no OS events in RCB-I patients.

## Discussion

In this study, we examined EOT ctDNA status and its combined use with RCB class as prognostic markers in TNBC patients with residual disease after NAST. ctDNA positivity was noted in 33% of patients, and ctDNA status was highly correlated with RCB class. We examined the association of EOT ctDNA status with outcomes in TNBC patients with residual disease after NAST and evaluated the combined use of ctDNA status and RCB class in this population.

Our ctDNA positivity rate and 3-year EFS and OS are in line with previous studies^8–10^. Though we observed favorable 3-year EFS and OS in ctDNA− patients compared to ctDNA+ patients, the fact that approximately 15–20% of ctDNA− patients do experience an EFS event indicates that ctDNA status alone likely cannot be used to recommend against adjuvant therapy in TNBC patients with residual disease. The RCB model was originally developed on a cohort of 241 patients treated with neoadjuvant chemotherapy^12^, and it has since been validated in numerous independent cohorts as a robust measure of long-term recurrence risk in breast cancer patients treated with NAST^6,7,13^. RCB is highly prognostic in TNBC, with 5-year relapse-free survival rates of approximately 94%, 89%, 62%, and 26% in patients with RCB-0 (pathologic complete response (pCR)), RCB-I, RCB-II, and RCB-III disease, respectively^6^. Despite its excellent performance, RCB is not a perfect model for recurrence risk and can be improved upon to aid in personalized decisions for an individual patient. The distribution of RCB classes in our study is quite similar to the distribution among 1004 TNBC patients with residual disease that was recently reported by Yau et al.^7^. We show that for RCB II patients, incorporating ctDNA status can identify a group that has 3-year EFS > 90%, for whom escalation of adjuvant systemic therapy with additional investigational agents may not yield a clinically meaningful survival benefit. More than two-thirds of TNBC patients with the residual disease have RCB-I/II, so this observation, if validated in other studies, can have potential clinical implications for a large proportion of patients. Similarly, the observation that 50% of patients with RCB-III disease who were ctDNA- did not experience an EFS event suggests that even the presence of a large amount of residual disease is not an absolute indicator of the eventual development of metastatic disease. On the other hand, the presence of both RCB-III disease and ctDNA+ status was associated with very dismal outcomes. These hypothesis-generating findings from our study regarding complementary prognostic information of ctDNA status and RCB should be confirmed in other data sets.

The observation that 30–40% of TNBC patients with residual disease who receive adjuvant systemic therapy develop recurrent disease^4,5^ highlights the urgent need to develop more effective adjuvant treatments for high-risk patients. Robust prognostic and predictive biomarkers of individual patients’ recurrence risk and therapeutic vulnerabilities will enable risk-adapted and personalized adjuvant therapy approaches, thus maximizing the efficacy of therapy and minimizing both physical and financial toxicity from unnecessary or ineffective therapy. Assessing novel therapeutic agents in the adjuvant setting for TNBC patients with the residual disease is an active area of investigation, and our results suggest that incorporating RCB class and ctDNA status into the design of these trials may improve trial efficiency.

Two recent experiences have reported on the utility of ctDNA as a prognostic marker in TNBC patients with residual disease after NAST. Radovich et al. analyzed 196 patients who were prospectively enrolled in the BRE12-158 study that randomized TNBC patients with residual disease after NAST to treatment of physician’s choice versus genomically-directed therapy. This study noted detectable ctDNA in 64% of patients and demonstrated that the presence of detectable ctDNA in patients with residual disease after NAST was associated with significantly inferior disease-free survival, distant-disease-free survival, and OS^9,14^. Similarly, an analysis of 22 TNBC patients enrolled in the I-SPY2 trial demonstrated that post-NAST (but before surgery) ctDNA was detectable in 14% of patients and was associated with an increased risk of distant recurrence^8^. The significant difference in ctDNA positivity rates in these two studies, despite both being sampled after neoadjuvant systemic therapy, may be explained by differences in methodology and ctDNA assessment time-point. ctDNA assessment in BRE12–158 was via a standardized multiplex panel (FoundationACT or FoundationOne), while in I-SPY2 it was based on an assessment of 12–16 personalized somatic mutations detected in patients’ primary tumors. In both BRE12-158 and I-SPY2 experiences, ctDNA assessment was performed before patients had received all definitive therapy. A recent small study of 11 TNBC patients with residual disease after NAST who required adjuvant radiotherapy reported that 3/11 (27%) patients had detectable ctDNA prior to radiotherapy, and that detection of cancer-associated variant alleles in plasma decreased dramatically in 2/3 (67%) of these patients during and after radiotherapy^11^. Though the small size of this study precludes any definitive conclusions, it demonstrates that EOT ctDNA assessment may be fundamentally more informative than an assessment that occurs before patients have completed all definitive therapy. It is possible that standard-of-care adjuvant systemic therapy (i.e., capecitabine or pembrolizumab) may also influence ctDNA status, and ongoing studies are evaluating serial ctDNA status in early-stage TNBC patients receiving adjuvant capecitabine (NCT04768426). Our study differs from BRE12–158 and I-SPY2 in that we assessed patients at the end of all curative therapy. Importantly, 3-year EFS and OS for our study population are in line with what is expected for contemporary patients when all degrees of residual disease are considered.

The goal of adjuvant treatment intensification is to eradicate occult micrometastatic disease. In our study, the median interval from detecting ctDNA to an EFS event was 4.7 months and most of the EFS events (81%) involved distant recurrence, suggesting that ctDNA positivity is a harbinger of rapid clinical disease recurrence. Our observation is similar to findings recently reported from the c-TRAK TN trial, which identified that ctDNA positivity was associated with a 4.1-month lead time until clinical detection of recurrence^10^. This is a clinically meaningful observation, as it has implications for the sequence of adjuvant systemic and local therapies and the design of trials incorporating adjuvant systemic therapy for TNBC patients with residual disease. Most patients in the CREATE-X trial^4^ completed radiotherapy before initiating capecitabine, and the current National Comprehensive Cancer Network (NCCN) guidelines indicate that capecitabine should be given after completion of radiotherapy (NCCN Version 2.2022). The short interval between the detection of ctDNA and clinical recurrence suggests that the window of opportunity for adjuvant systemic therapy to eradicate non-locoregional micrometastatic disease may be very small. The findings from our study and the cTRAK TN trial suggest that future investigations might consider swift initiation of adjuvant systemic therapy prior to radiotherapy in patients at a very high risk of distant recurrence, as that risk might significantly outweigh the risk of locoregional recurrence.

Our study has several limitations. First, ctDNA status was assessed retrospectively in archival plasma samples, though the collection of all blood and clinical follow-up data was performed prospectively as part of our ongoing registry (PROGECT; NCT02302742). We also did not have radiographic staging performed concurrently with plasma collection, and recent data suggest that many patients with detectable ctDNA, especially those with residual nodal disease after NAST, may have overt metastatic disease^10^. An additional limitation is that the ctDNA assessment was only performed at a single time point, and this occurred over a period of six months. ctDNA status may be a dynamic biomarker, with ctDNA+ patients converting to ctDNA- as a consequence of adjuvant therapy^11^, or ctDNA− patients converting to ctDNA+ on later assessments^10^. We also did not have comprehensive germline sequencing available for these patients, though we were able to corroborate the majority of 40–60% VAF cfDNA mutations as germline, and/or the presence of a 40–60% VAF cfDNA mutation did not change their status, since nearly all of those patients had co-existing low-frequency cfDNA mutation(s) that otherwise met the criteria for ctDNA+ status. Interestingly, in the single patient with a detectable 40–60% VAF cfDNA *BRCA2* mutation who did not undergo germline genetic testing, we also found co-existing 40–60% VAF cfDNA mutations in *SRSF2* and *STAG2*. Both mutations are seen commonly in patients with myelodysplastic syndrome (MDS)^15,16^, and this patient did receive a subsequent diagnosis of MDS. We suspect that this patient carried a germline mutation in *BRCA2*, which is known to increase the risk of myeloid malignancy^17^, and that the *SRSF2* and *STAG2* cfDNA mutations were arising from dysplastic myeloblasts rather than her TNBC.

All patients in our study had residual disease so we are unable to report on the impact of ctDNA status on prognosis in patients with pCR. Neoadjuvant treatment among the 80 patients in our series varied, with most patients receiving standard chemotherapy treatment of physician’s choice, and some patients (23%) receiving chemotherapy plus immunotherapy on a clinical trial (NCT03639948). Though this heterogeneous treatment and 6-month collection period limit the interpretation of our results in the context of a defined systemic therapy regimen or defined post-treatment timepoint, they reflect real-world practice. The number of patients who received neoadjuvant immunotherapy is small and precludes a subset analysis of ctDNA status within this population, but the inclusion of these patients is relevant given the recent approval of neoadjuvant pembrolizumab with chemotherapy in high-risk early-stage TNBC patients^18^. It is important to interpret our findings accordingly, since most TNBC patients will now receive immunotherapy in the neoadjuvant and adjuvant setting, and data suggest that ctDNA dynamics during treatment with pembrolizumab are prognostic in breast cancer^19^. Lastly, we used a non-personalized sequencing methodology that assesses mutations in 275 cancer-related genes, and we dichotomized ctDNA status based on an internally optimal lower boundary VAF threshold. This leads to inherent bias and highlights the need to validate our methodology in an independent dataset. Tumor-informed approaches that interrogate ctDNA for discrete alterations that are known to exist in the primary tumor may lead to improved sensitivity and specificity. Despite the limitations of our study, we demonstrate that end-of-treatment ctDNA status is highly prognostic and that its combined use with RCB class provides better prognostication than either biomarker alone.

## Methods

### Patients and samples

The study population included patients with stage I-III TNBC (defined as estrogen receptor (ER) ≤ 10%, progesterone receptor (PR) ≤ 10%, and negative for human epidermal growth factor receptor 2 (HER2) by ASCO-CAP criteria^20^) who were enrolled on an IRB-approved multisite prospective registry (PROGECT; NCT02302742) between 2011 and 2020 and had residual disease after NAST with available EOT plasma samples. EOT plasma samples were collected 1–6 months after completion of all curative treatment (surgery, chemotherapy, and/or radiotherapy, whichever ended last). RCB was calculated using defined clinicopathologic features as previously described^12^. Demographic, clinical, pathologic, and treatment information was collected (Table 1), and participants were prospectively followed for recurrence and survival. Patients received systemic and locoregional treatment as per the recommendations of their treating physicians. All patients provided written informed consent, and the study was approved by the institutional review board at the University of Kansas Medical Center.

### Blood collection, processing, and storage

Blood was collected into acid-citrate dextrose (ACD) blood tubes and processed within four hours of collection using standard blood processing methods. Briefly, whole blood was centrifuged once at 1300 × *g* for 10 min in a swinging bucket centrifuge with the brakes turned off. The upper plasma was collected without disrupting the buffy coat layer, aliquoted (1 mL/aliquot) into prelabeled cryovials, and stored frozen at −80 °C. Cell-free DNA was isolated from 2 mL of ACD plasma using the QIAamp Circulating Nucleic Acid kit (Qiagen) following the manufacturer’s protocol for the vacuum manifold method and then sequenced.

### ctDNA sequencing

ctDNA quality was assessed by TapeStation (Agilent) and then concentrated approximately three-fold using a Savant DNA SpeedVac on the no-heat setting. Libraries were prepared from concentrated ctDNA using the QIAseq Targeted DNA Human Comprehensive Cancer Panel (Qiagen) which targets exons of 275 genes. The quality and concentration of the prepared libraries were assessed by TapeStation. Equimolar libraries were pooled (~16–19 samples/pool) and 151-cycle paired-end sequencing was performed on an Illumina NextSeq 550 instrument using a high-output flow cell. Raw sequence data in FASTQ format were processed through CLC Genomics Workbench (Qiagen) against GRCh37 to generate variant call format (vcf) files as well as sequencing quality metrics. Patients with mutations only in *DNMT3A*, *TET2*, *ASXL1*, or *JAK2* were classified as ctDNA- as these mutations likely arise from clonal hematopoiesis of indeterminate potential (CHIP)^21^. Patients with detectable pathogenic or likely pathogenic variants (variant allelic frequency (VAF) ≥ 3.0% but <40% or >60%) were considered ctDNA+. When possible, mutations detected with a frequency of ≥40% were corroborated as germline by referencing clinical genetic testing reports. Patients with one or more mutation(s) detected at 40–60% frequency, confirmed or unconfirmed germline, without any mutation(s) detected in the ≥3.0% but <40% or >60% range were classified as ctDNA−. Patients with one or more mutation(s) detected in the ≥3.0% but <40% or >60% range were classified as ctDNA+ (unless all confirmed germline), regardless of whether additional mutations at 40–60% were detected.

### Sample size and statistical analysis

Baseline characteristics were compared across groups by chi-squared or Fisher’s exact tests, with Mann–Whitney *U* test used for continuous variables. Event-free survival (EFS) and overall survival (OS) were estimated according to the Kaplan-Meier method and compared across groups by log-rank test, followed by Cox regression analysis. EFS was defined as the time from diagnosis to first recurrence (invasive ipsilateral breast, invasive local/regional, or distant), or to death from breast cancer. OS was defined as the time from diagnosis to death from any cause. Patients were censored on the date of last contact if an event had not been observed. All reported *P* values and confidence intervals (CI) are from two-sided tests. *P* values <0.05 were considered statistically significant. All analyses were conducted using SPSS Statistics version 27 (IBM Corporation).

With an anticipated ctDNA positivity rate of 50% and 3-year event-free survival of 55% in ctDNA+ patients and 80% in ctDNA− patients based on previously published data^8,9^, we projected that we would need a minimum sample size of 60 patients to have 86% power to detect this 25% difference in 3-year EFS between ctDNA− and ctDNA+ negative and positive patients with a one-sided *α* = 0.05. Because of the uncertainty in the ctDNA positivity rate and effect size, we elected to sequence 80 samples to ensure adequate statistical power.

### Reporting summary

Further information on research design is available in the Nature Research Reporting Summary linked to this article.

## Supplementary information

Reporting Summary

Supplementary Figures

## Data Availability

The datasets generated and analyzed during the current study are not publicly available due to them containing information that could compromise research participant privacy. Additionally, explicit consent to deposit participant-level data was not obtained from participants, and many participants are deceased or lost to follow-up, which precludes obtaining consent for the data deposition. However, a limited set of de-identified data can be made available by the corresponding author (P.S.) upon reasonable request.

## References

[1] Li X (2017). Triple-negative breast cancer has worse overall survival and cause-specific survival than non-triple-negative breast cancer. Breast Cancer Res. Treat..

[2] Liedtke C (2008). Response to neoadjuvant therapy and long-term survival in patients with triple-negative breast cancer. J. Clin. Oncol..

[3] Spring LM (2020). Pathologic complete response after neoadjuvant chemotherapy and impact on breast cancer recurrence and survival: a comprehensive meta-analysis. Clin. Cancer Res.

[4] Masuda N (2017). Adjuvant capecitabine for breast cancer after preoperative chemotherapy. N. Engl. J. Med..

[5] Mayer IA (2021). Randomized phase III postoperative trial of platinum-based chemotherapy versus capecitabine in patients with residual triple-negative breast cancer following neoadjuvant chemotherapy: ECOG-ACRIN EA1131. J. Clin. Oncol..

[6] Symmans WF (2017). Long-term prognostic risk after neoadjuvant chemotherapy associated with residual cancer burden and breast cancer subtype. J. Clin. Oncol..

[7] Yau C (2022). Residual cancer burden after neoadjuvant chemotherapy and long-term survival outcomes in breast cancer: a multicentre pooled analysis of 5161 patients. Lancet Oncol..

[8] Magbanua MJM (2021). Circulating tumor DNA in neoadjuvant-treated breast cancer reflects response and survival. Ann. Oncol..

[9] Radovich M (2020). Association of circulating tumor DNA and circulating tumor cells after neoadjuvant chemotherapy with disease recurrence in patients with triple-negative breast cancer: preplanned secondary analysis of the BRE12-158 randomized clinical trial. JAMA Oncol..

[10] Turner, N. et al. in *San Antonio Breast Cancer Symposium*.

[11] Kim H (2021). Dynamics of circulating tumor DNA during postoperative radiotherapy in patients with residual triple-negative breast cancer following neoadjuvant chemotherapy: a prospective observational study. Breast Cancer Res. Treat..

[12] Symmans WF (2007). Measurement of residual breast cancer burden to predict survival after neoadjuvant chemotherapy. J. Clin. Oncol..

[13] Symmans WF (2021). Assessment of residual cancer burden and event-free survival in neoadjuvant treatment for high-risk breast cancer: an analysis of data from the I-SPY2 randomized clinical trial. JAMA Oncol..

[14] Schneider, B. P. et al. BRE12-158: a postneoadjuvant, randomized phase II trial of personalized therapy versus treatment of physician’s choice for patients with residual triple-negative breast cancer. *J. Clin. Oncol.*10.1200/JCO.21.01657 (2021). 10.1200/JCO.21.01657 34910554

[15] Thota S (2014). Genetic alterations of the cohesin complex genes in myeloid malignancies. Blood.

[16] Wu SJ (2012). The clinical implication of SRSF2 mutation in patients with myelodysplastic syndrome and its stability during disease evolution. Blood.

[17] Friedenson B (2007). The BRCA1/2 pathway prevents hematologic cancers in addition to breast and ovarian cancers. BMC Cancer.

[18] Schmid P (2020). Pembrolizumab for early triple-negative breast cancer. N. Engl. J. Med..

[19] Bratman SV (2020). Personalized circulating tumor DNA analysis as a predictive biomarker in solid tumor patients treated with pembrolizumab. Nat. Cancer.

[20] Wolff AC (2018). Human epidermal growth factor receptor 2 testing in breast cancer: American Society of Clinical Oncology/College of American Pathologists Clinical Practice Guideline Focused Update. Arch. Pathol. Lab. Med..

[21] Buscarlet M (2017). DNMT3A and TET2 dominate clonal hematopoiesis and demonstrate benign phenotypes and different genetic predispositions. Blood.

