# Supplementary material for: ctDNA and residual cancer burden are prognostic in triple-negative breast cancer patients with residual disease
Source: NPJ Breast Cancer. 2023 Mar 6;9:10. doi: 10.1038/s41523-023-00512-7 (PMC9988835; doi:10.1038/s41523-023-00512-7)
Supplement: Supplementary file 1 — Reporting Summary [file 41523_2023_512_MOESM1_ESM.pdf]

nature portfolio

Corresponding author(s): Priyanka Sharma

Last updated by author(s): Jan 18, 2023

Reporting Summary

Nature Portfolio wishes to improve the reproducibility of the work that we publish. This form provides structure for consistency and transparency in reporting. For further information on Nature Portfolio policies, see our Editorial Policies and the Editorial Policy Checklist.

Statistics

For all statistical analyses, confirm that the following items are present in the figure legend, table legend, main text, or Methods section.

n/a	Confirmed	
☐	☒	The exact sample size (*n*) for each experimental group/condition, given as a discrete number and unit of measurement
☐	☒	A statement on whether measurements were taken from distinct samples or whether the same sample was measured repeatedly
☐	☒	The statistical test(s) used AND whether they are one- or two-sided<br>*Only common tests should be described solely by name; describe more complex techniques in the Methods section.*
☐	☒	A description of all covariates tested
☒	☐	A description of any assumptions or corrections, such as tests of normality and adjustment for multiple comparisons
☐	☒	A full description of the statistical parameters including central tendency (e.g. means) or other basic estimates (e.g. regression coefficient) AND variation (e.g. standard deviation) or associated estimates of uncertainty (e.g. confidence intervals)
☐	☒	For null hypothesis testing, the test statistic (e.g. *F*, *t*, *r*) with confidence intervals, effect sizes, degrees of freedom and *P* value noted<br>*Give P values as exact values whenever suitable.*
☒	☐	For Bayesian analysis, information on the choice of priors and Markov chain Monte Carlo settings
☒	☐	For hierarchical and complex designs, identification of the appropriate level for tests and full reporting of outcomes
☒	☐	Estimates of effect sizes (e.g. Cohen's *d*, Pearson's *r*), indicating how they were calculated

Our web collection on statistics for biologists contains articles on many of the points above.

Software and code

Policy information about availability of computer code

Data collection	Data were collected and maintained in a secure RedCap database
Data analysis	IBM SPSS Statistics, Version 27

For manuscripts utilizing custom algorithms or software that are central to the research but not yet described in published literature, software must be made available to editors and reviewers. We strongly encourage code deposition in a community repository (e.g. GitHub). See the Nature Portfolio guidelines for submitting code & software for further information.

Data

Policy information about availability of data

All manuscripts must include a data availability statement. This statement should provide the following information, where applicable:

- Accession codes, unique identifiers, or web links for publicly available datasets
- A description of any restrictions on data availability
- For clinical datasets or third party data, please ensure that the statement adheres to our policy

The datasets generated and analyzed during the current study are not publicly available due to them containing information that could compromise research participant privacy. Additionally, explicit consent to deposit participant-level data was not obtained from participants, and many participants are deceased or lost to follow-up, which precludes obtaining consent for the data deposition. However, a limited set of de-identified data can be made available by the corresponding author (P.S.) upon reasonable request.

March 2021

Human research participants

Policy information about studies involving human research participants and Sex and Gender in Research.

Reporting on sex and gender	While 1% of all breast cancers arise in males, TNBC in male patients is exceptionally rare. Though male breast cancer patients are eligible to enroll in the PROGECT registry, only female patients were identified for this study.
Population characteristics	The study population included patients with stage I-III TNBC (defined as estrogen receptor (ER) <10%, progesterone receptor (PR) <10%, and negative for human epidermal growth factor receptor 2 (HER2) by ASCO-CAP criteria.
Recruitment	Participants with TNBC who were evaluated and/or treated for TNBC at the University of Kansas Cancer Center were offered voluntary enrollment in the PROGECT prospective registry.
Ethics oversight	All patients provided written informed consent, and the study was approved by the institutional review board at the University of Kansas Medical Center.

Note that full information on the approval of the study protocol must also be provided in the manuscript.

Field-specific reporting

Please select the one below that is the best fit for your research. If you are not sure, read the appropriate sections before making your selection.

☒ Life sciences ☐ Behavioural & social sciences ☐ Ecological, evolutionary & environmental sciences

For a reference copy of the document with all sections, see nature.com/documents/nr-reporting-summary-flat.pdf

Life sciences study design

All studies must disclose on these points even when the disclosure is negative.

Sample size	With an anticipated ctDNA positivity rate of 50% and 3-year event free survival of 55% in ctDNA+ patients and 80% in ctDNA- patients based on previously published data,9 we projected that we would need a minimum sample size of 60 patients to have 86% power to detect this 25% difference in 3-year EFS between ctDNA negative and positive patients with a one-sided α=0.05.
Data exclusions	Not applicable.
Replication	Previous studies have shown an association between ctDNA and outcome and RCB class and outcome. Our findings provide additional insight in to the role of end-of-treatment ctDNA status and the combined utility of ctDNA status and RCB in predicting outcomes in TNBC patients with residual disease.
Randomization	We analyzed variant allele frequency (VAF) thresholds from 1-5% to define ctDNA positivity. A 3% threshold was chosen to dichotomize patients in to ctDNA- and ctDNA+ categories as this gave the best discrimination in terms of actuarial event-free survival.
Blinding	This was a retrospective analysis ctDNA and RCB with outcomes among patients enrolled in a prospective observational trial. Blinding was not possible.

Reporting for specific materials, systems and methods

We require information from authors about some types of materials, experimental systems and methods used in many studies. Here, indicate whether each material, system or method listed is relevant to your study. If you are not sure if a list item applies to your research, read the appropriate section before selecting a response.

Materials & experimental systems

n/a	Involved in the study	
☒	☐	Antibodies
☒	☐	Eukaryotic cell lines
☒	☐	Palaeontology and archaeology
☒	☐	Animals and other organisms
☐	☒	Clinical data
☒	☐	Dual use research of concern

Methods

n/a	Involved in the study	
☒	☐	ChIP-seq
☒	☐	Flow cytometry
☒	☐	MRI-based neuroimaging

Clinical data

Policy information about clinical studies

All manuscripts should comply with the ICMJE guidelines for publication of clinical research and a completed CONSORT checklist must be included with all submissions.

Clinical trial registration	NCT02302742
Study protocol	Uploaded as an attachment along with the manuscript.
Data collection	Patients in this study were enrolled on an IRB-approved multisite prospective registry (PROGECT; NCT02302742) between 2011 and 2020 and had residual disease after NAST with available EOT plasma samples.
Outcomes	Outcomes of interest were event-free survival (EFS) and overall survival (OS), which were
