# Supplementary material for: ctDNA and residual cancer burden are prognostic in triple-negative breast cancer patients with residual disease
Source: NPJ Breast Cancer. 2023 Mar 6;9:10. doi: 10.1038/s41523-023-00512-7 (PMC9988835; doi:10.1038/s41523-023-00512-7)
Supplement: Supplementary file 2 — Supplementary Figures [file 41523_2023_512_MOESM2_ESM.pdf]

Supplementary Figure 1

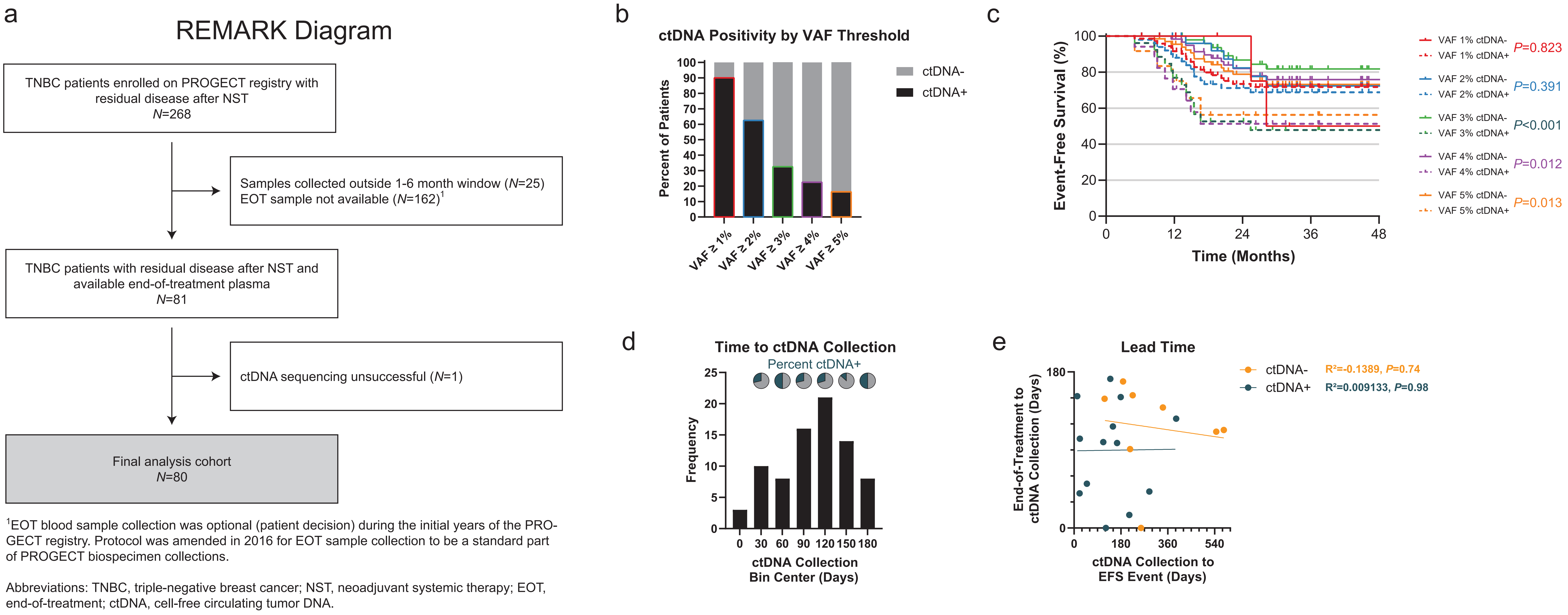

(a) REMARK diagram. (b) ctDNA positivity rates by VAF thresholds ranging from 1-5%. (c) Comparison of EFS by ctDNA status using VAF thresholds of 1-5%. *P* value is log-rank. (d) Histogram showing temporal distribution of end-of-treatment ctDNA collection. Pie charts show the percent of patients within that 30-day bin who were found to be ctDNA+. (e) Association between end-of-treatment to ctDNA assessment interval and ctDNA assessment to EFS event interval in amongst patients who experienced recurrence. R^2 and *P* reflect Pearson correlation coefficient and significance, respectively.

Supplementary Figure 2

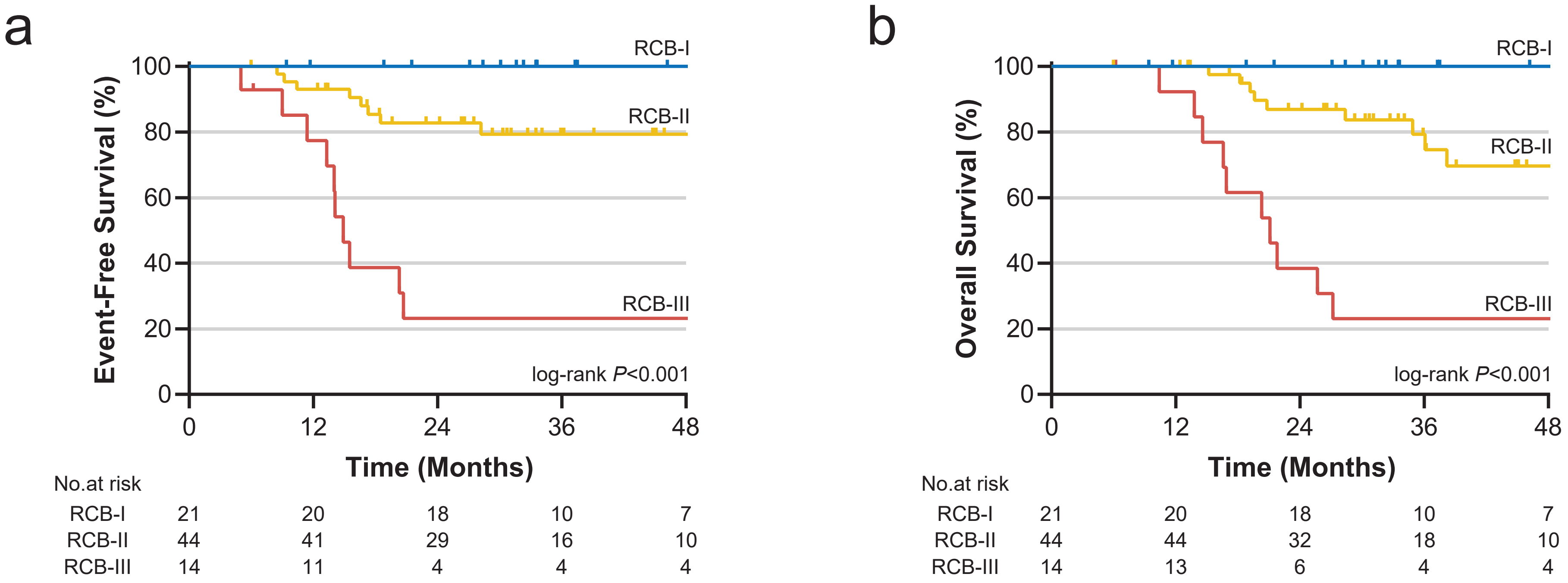

(a) EFS among RCB-I, RCB-II, and RCB-III classes. (b) OS among RCB-I, RCB-II, and RCB-III classes. *P* is log-rank statistic.
